# Two-Way Shape Memory Effect Induced by Tensile Deformation in Columnar-Grained Cu_71.7_Al_18.1_Mn_10.2_ Alloy

**DOI:** 10.3390/ma11112109

**Published:** 2018-10-26

**Authors:** Pei-Sheng Yao, Hai-You Huang, Yan-Jing Su, Jian-Xin Xie

**Affiliations:** 1 Key Laboratory for Advanced Materials Processing of the Ministry of Education, Institute for Advanced Materials and Technology, University of Science and Technology Beijing, Beijing 100083, China; beikeyaops@163.com (P.-S.Y.); jxxie@mater.ustb.edu.cn (J.-X.X.); 2 Beijing Advanced Innovation Center for Materials Genome Engineering, University of Science and Technology Beijing, Beijing 100083, China; yjsu@ustb.edu.cn

**Keywords:** Cu-Al-Mn, shape memory alloy, columnar grain, two-way shape memory effect, pre-deformation strain

## Abstract

Columnar-grained Cu_71.7_Al_18.1_Mn_10.2_ shape memory alloy (SMA) was prepared by a directional solidification method and exhibited a high superelasticity of 8.18% and excellent ductility at room temperature, which provided the possibility of obtaining high shape memory. However, proper pre-deformation is an essential part of repeatedly obtaining large and stable shape change. In this paper, one-time uniaxial tensile pre-deformation was carried out at the temperature range −70–−80 °C. Then, the two-way shape memory effect (TWSME) of the alloy was evaluated by the martensitic transformation strain (*ε*_M_) which was measured by a thermal expansion test to investigate the relationship between the pre-deformation strain (*ε*_T_) and the TWSME. The results showed that *ε*_M_ of the columnar-grained Cu_71.7_Al_18.1_Mn_10.2_ alloy increased at first and then decreased with the increase of *ε*_T_. The maximum value 2.91% of the *ε*_M_ could be reached when *ε*_T_ was 6%. The effects of the *ε*_T_ on transformation temperatures were also measured by differential scanning calorimetry. Based on the variations of transformation temperatures, the relationship between the internal stress induced by the pre-deformation process and the *ε*_M_, and the influence mechanism of the pre-deformation strain on the TWSME in columnar-grained Cu_71.7_Al_18.1_Mn_10.2_ alloy, were discussed. The results obtained from this work may provide reference for potential applications of Cu-based SMAs, such as self-control components, fasteners, etc.

## 1. Introduction

The two-way shape memory effect (TWSME) associated with the forward and reverse transformations of martensite is a phenomenon in which the reversible and spontaneous shape change during heating and cooling occur without any external stress [1]. The TWSME not only relates to the behavior of martensite transformation in shape memory alloys (SMAs), but also depends on training methods such as superelastic (SE) or shape memory effect (SME) training, thermomechanical training, aging under external stress, etc. [1] and the training parameters. One of the easiest training methods is thermomechanical training, which typically involves a mechanical cycle (deformation applied and then unloaded) at low temperature (below martensite transformation finish temperature, *M*_f_) which is often called cryogenic pre-deformation, and then restoring the original shape by subsequently increasing the temperature to a high temperature (above austenite transformation finish temperature, *A*_f_) without any external stress [2]. In this procedure, the deformation strain applied at low temperature is defined as the pre-deformation strain (*ε*_T_). If the uniaxial tensile loading is chosen for the cryogenic pre-deformation, the TWSME can be evaluated by the elongation induced by the martensite transformation strain (*ε*_M_) upon cooling or the contraction induced by the reverse/austenite transformation strain (*ε*_A_) upon heating. For a fully recovered deformation, the transformation strain for the thermal cycle (*ε*_TW_) can be used as *ε*_TW_ = *ε*_M_ = *ε*_A_.

During thermomechanical treatment of SMAs, much research indicated that the *ε*_T_ applied on the sample is usually 2–5 times as large as the *ε*_TW_ finally obtained [1,3,4,5]. For example, *ε*_TW_ of 4.1% and 2% were obtained in Ni–Ti alloy and Cu–Zn–Al alloy after pre-deformation with *ε*_T_ of 13.2% and 7%, respectively [3,4]. Hence, good ductility may be a precondition for a high *ε*_TW_ in SMAs, which could guarantee a sufficient *ε*_T_ to apply onto the SMAs without cracking or fracturing. For Cu-based SMAs, single crystal alloys exhibit good ductility [6,7]. Nevertheless, single crystal Cu-based SMAs are still limited in many potential applications due to the high preparation costs and the difficulty in the preparation of large-sized, single, crystalline bulk [6]. Meanwhile, ordinary polycrystalline Cu-based SMAs, due to their high elastic anisotropy (elastic anisotropy factor of 12–13 on austenite phase [8], while on Ni–Ti alloys only ~2 [9]) and large martensitic transformation strain anisotropy [10] which leads to serious deformation and transformation incompatibility between different grains with random orientations, always suffered from intergranular brittle fracture and low ductility. Hence, ordinary polycrystalline Cu-based SMAs show a small apparent *ε*_TW_ due to their low ductility. The development of ductile polycrystalline Cu-based SMAs becomes a key to realizing wide industrial application. In the 1990s, Kainuma et al. developed ductile Cu–Al–Mn alloys with a high Mn content of about 10 at.% and a low Al content of 16–18.5 at.% due to an appropriate decrease of the degree of order in the L2_1_ austenite phase, in which the martensite phase has the 18R structure [11,12]. Omori et al. evaluated the TWSME of the ductile polycrystalline Cu–Al–Mn alloys using a bending pre-deformation method. A maximum value of *ε*_TW_ = 3.2% was obtained after ~12.5% pre-deformation strain [1,12]. For further improvement on the ductility and SME properties of Cu–Al–Mn alloys, Omori et al. [13] and Liu et al. [14,15] prepared bamboo-like-grained and columnar-grained alloys respectively by microstructure control. The mechanical properties of the polycrystalline alloys can be promoted to the single crystal level due to these microstructure characteristics of highly concentrated grain orientation and large grain size.

On the other hand, improving pre-deformation efficiency (the value of *ε*_TW_/*ε*_T_) is another way to obtain higher *ε*_TW_ under a certain pre-deformation strain. Considering the energy dissipation, minimization of the energy dissipation induced by microstructure defects, grain boundaries, etc. during thermomechanical pre-deformation of SMAs, could increase the pre-deformation efficiency [16]. In our previous work, columnar-grained Cu–Al–Mn alloys with <001>-oriented texture and a large grain size were prepared by a directional solidification method and showed many significant features such as high transformation and deformation compatibility, low transformation resistance and internal friction [14]. A high superelasticity of above 8% and high ductility of above 40% were measured in the columnar-grained Cu_71_Al_18_Mn_11_ alloys at room temperature [14], which provides a great prerequisite for high TWSME, while ordinary polycrystalline Cu–Al–Mn alloys exhibit a low superelasticity of only ~4% [17]. Therefore, the purpose of this work is to explore the TWSME in columnar-grained Cu–Al–Mn alloys. The effect of pre-deformation strain on the *ε*_TW_ in columnar-grained Cu–Al–Mn alloys was experimentally investigated. The internal stresses induced by tensile pre-deformation were also analyzed by transformation temperature measurement to discuss the effect mechanism of pre-deformation strain on the TWSME of columnar-grained Cu–Al–Mn alloys.

## 2. Experimental Procedure

The raw materials used in our experiments were oxygen-free copper (99.95 wt.% purity), electrolytic aluminum (99.99 wt.% purity) and electrolytic manganese (99.9 wt.% purity), which were cast into precast ingots by vacuum induction melting. Subsequently, the precast ingots were re-melted and directionally solidified to obtain columnar-grained ingots with a dimension of Φ50 × 150 mm [14]. The ingots were homogenized at 800 °C for 5 h and water-quenched to room temperature. Finally, an aging treatment was carried out at 150 °C for 5 h to stabilize the martensitic transformation temperature. About 50 mg debris cut from the middle part of the ingot was determined by OPTIMA 7000 DV (PerkinElmer Instruments Company, San Antonio, TX, USA) inductively coupled plasma spectroscopy (ICP) to chemical compositions analyses. The results showed that the actual compositions of the alloy were Cu:Al:Mn = 71.7:18.1:10.2 (at.%). The martensitic transformation temperatures were measured by Q2000 V24.11 Build 124 (Mettle Toledo Company, Greifensee, Switzerland) differential scanning calorimetry (DSC) at a heating and cooling rate of 10 °C/min.

The metallographic samples were cut from the middle part of the ingot by electrical-discharge machining, which was observed by Nikon Eclipse LV150 optical microscope (Nikon Instruments Company, Tokyo, Japan) after being mechanically polished and etched by a FeCl_3_ hydrochloric solution. The phase and microstructure were analyzed by Rigaku D/MAX-RB12KW X-ray diffraction (XRD, Rigaku Company, Tokyo, Japan) with Cu *K*α radiation and a Tecnai G2 F20 field emission transmission electron microscope (TEM, FEI Company, Fremont, CA, USA). The orientation of the grains was analyzed by electron backscatter diffraction (EBSD).

The SE test and cryogenic pre-deformation experiments were conducted using dog-bone-shaped samples with a gauge length of 30 mm and a section area of 8 × 3 mm^2^. During deformation a local strain was measured using an Instron extensometer with a gauge length of 25 mm. The longitudinal direction of the samples was parallel to the solidification direction (SD). Superelasticity was examined by a cycle tensile test with a strain rate of 5 × 10^−4^ s^−1^ at room temperature in an austenite phase state [18], that is a series of uniaxial tensile loading and unloading were performed using one specimen with increasing strain amplitude of 2%.

The cryogenic pre-deformation experiment was conducted by Instron universal mechanical test systems with a thermostatic chamber, and nine specimens were used to load to 2%, 3%, 4%, 5%, 6%, 8%, 10%, 12%, 14%, respectively. All cryogenic pre-deformation specimens were cut from the same ingot to avoid the effects of deviation from the composition and casting processes on the comparability of test results. The detailed procedure of cryogenic pre-deformation experiment was as follows. First, the cryogenic pre-deformation samples were cooled to −70–−80 °C into martensite phase state for 1 h using liquid nitrogen (the temperature was manually controlled and monitored by a T-type thermocouple with a sampling frequency of 40 Hz and an accuracy of 1 °C). The cryogenic pre-deformation samples in the martensite phase state were loaded to a specific strain with a strain rate of 5 × 10^−4^ s^−1^ in a nearly isothermal condition, held for 300 s, and then unloaded at a strain rate of 1 × 10^−4^ s^−1^. Subsequently, the cryogenic pre-deformation samples were taken into ambient temperature and *L*_0_ = 20 mm rectangular plate samples for the thermal expansion test were cut from the middle of the cryogenic pre-deformation samples with a 25 mm gauge length and examined by a laser thermal expansion instrument (LINSEIS L75, LINSEIS Instrument and Equipment Company, Selb, Germany) with an accuracy of 1 × 10^−3^ mm at a heating and cooling rate of 2 °C/min. It should be noted that during the thermal expansion measurement, a very small pressure of about 0.1–1 N was applied to the samples.

## 3. Results and Discussion

### 3.1. Transformation Temperatures and Microstructure of Directional Solidification Cu_71.7_Al_18.1_Mn_10.2_ SMAs

**Figure 1:**
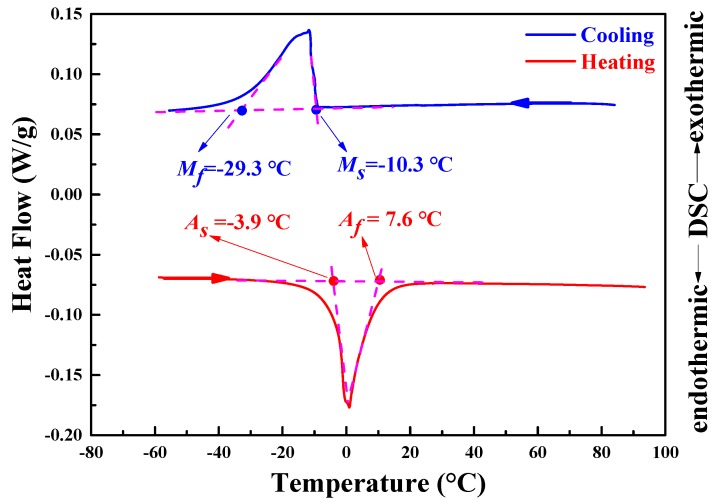
DSC curves and transformation temperatures of Cu_71.7_Al_18.1_Mn_10.2_ alloy sample. Note: *M*_s_ is the martensite transformation starting temperature. *M*_f_ is the martensite transformation finishing temperature. *A*_s_ is the austenite transformation starting temperature. *A*_f_ is the austenite transformation finishing temperature.

The DSC curves of the Cu_71.7_Al_18.1_Mn_10.2_ sample are shown in Figure 1. Transformation temperatures were determined from the DSC curve to be the martensite transformation starting temperature *M*_s_ = −10.3 °C, the martensite transformation finish temperature *M*_f_ = −29.3 °C, the austenite transformation starting temperature *A*_s_ = −3.9 °C, and the austenite transformation finish temperature *A*_f_ = 7.6 °C. The transformation heat was also measured based on the DSC curves as 5.7 J/g for the martensitic transformation on cooling and 6.5 J/g for the reverse transformation on heating.

**Figure 2:**
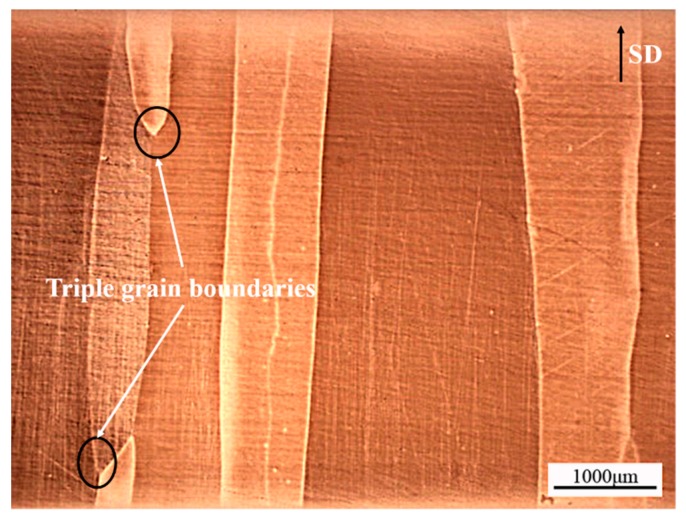
The optical microscopy graph of Cu_71.7_Al_18.1_Mn_10.2_ alloy sample prepared by directional solidification. SD: Solidification direction.

**Figure 3:**
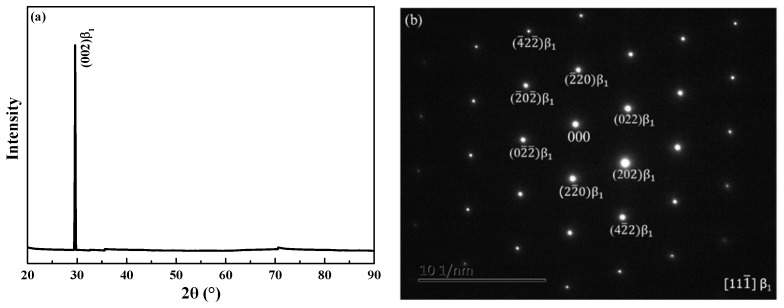
(**a**) XRD profile; (**b**) TEM diffraction pattern of columnar-grained Cu_71.7_Al_18.1_Mn_10.2_ alloy sample.

Figure 2 shows that the columnar grains grow parallel to the SD with straight grain boundaries. There were almost no transverse grain boundaries and only a small amount of triple grain boundaries. The average width of the columnar grains was 1.26 mm. The columnar-grained Cu_71.7_Al_18.1_Mn_10.2_ alloy sample was composed of a single *β*_1_ phase (austenite phase) at room temperature as proved by XRD (Figure 3a). The crystallographic parameters of the bcc *β*_1_ phase were detected by the XRD and TEM diffraction pattern as being *a* = *b* = *c* = 0.572 nm, *α* = *β* = *γ* = 90^o^ as shown in Figure 3a,b.

**Figure 4:**
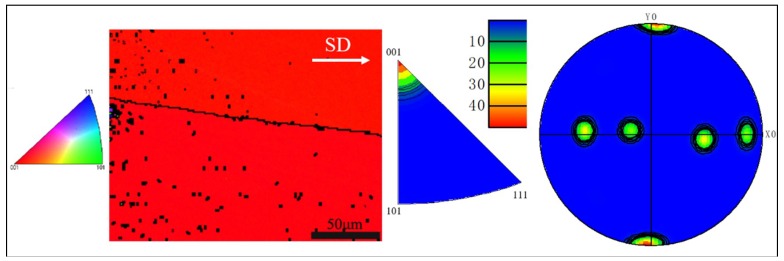
Morphology, inverse pole figure and <001> pole figure of columnar-grained Cu_71.7_Al_18.1_Mn_10.2_ alloy sample. SD: Solidification direction.

It could be seen from the morphology in Figure 4 that columnar-grained Cu_71.7_Al_18.1_Mn_10.2_ alloy had almost no sub-grain boundaries. The columnar grains’ orientation was distributed near <001> as we were able to see from the inverse pole figure and pole figure in Figure 4, which illustrated that the sample had a strong <001>-oriented texture along the SD.

### 3.2. Pre-Deformation and TWSME of Columnar-Grained Cu_71.7_Al_18.1_Mn_10.2_ SMA

**Figure 5:**
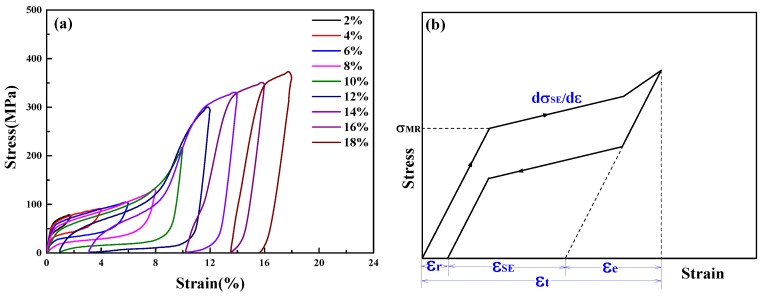
(**a**) SE cyclic stress-strain curves of a columnar-grained Cu_71.7_Al_18.1_Mn_10.2_ alloy sample tested at room temperature; (**b**) sketch map of property parameters based on SE cyclic stress–strain curves.

The SE cyclic stress-strain curve tested at room temperature was plotted in Figure 5a. The sample was successively loaded and unloaded with a strain step of 2% to a maximum total strain (*ε*_t_) of 18%. With the increase of cycles and total strain, the SE strain (*ε*_SE_) increased at first and then decreased, where *ε*_SE_ *= ε*_t_ − *ε*_r_ − *ε*_e_, *ε*_r_ was unrecovered strain and *ε*_e_ was elastic strain, as shown in Figure 5b. When the *ε*_t_ was up to 10%, the maximum *ε*_SE_ was 8.18% (Figure 5a). The critical stress of the stress-induced martensitic transformation (*σ*_M_) at the first cyclic strain was about 60 MPa and decreased with the increase in the number of cycles. The stress plateau slope d*σ*_SE_/d*ε* of the stress induced martensitic transformation stage was 7.3 MPa. The cyclic stress-strain curves showed that the columnar-grained Cu_71.7_Al_18.1_Mn_10.2_ alloy exhibited excellent superelasticity along the SD. The normal tensile test was also conducted and showed that the elongation reached more than 40%, which indicated a high ductility of the columnar-grained Cu_71.7_Al_18.1_Mn_10.2_ alloy sample.

**Figure 6:**
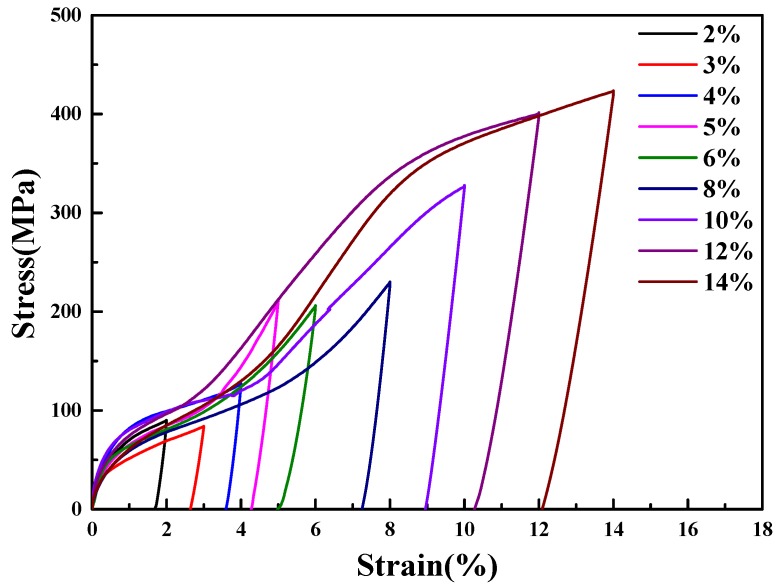
The tensile stress–strain curves of columnar-grained Cu_71.7_Al_18.1_Mn_10.2_ alloy samples during cryogenic pre-deformation.

The cryogenic pre-deformation tensile curves of 2%, 3%, 4%, 5%, 6%, 8%, 10%, 12%, 14% strains respectively for nine columnar-grained Cu_71.7_Al_18.1_Mn_10.2_ alloy samples extended at −70–−80 °C (<*M*_s_ −50 °C) are shown in Figure 6. During the pre-deformation process, the sample was kept in a martensite state. With the increase in loading strain, the reorientation of martensitic variants occurred after the elastic deformation and made a “yield” phenomenon appear in the stress-strain curve, where the “yield” stress was often called critical stress of martensite reorientation (*σ*_MR_) [19]. Furthermore, the reorientation of martensitic variants continued to finish and the dislocation glide started. According to Figure 6, there was not a sharp boundary between the reorientation of martensitic variants and the dislocation glide. Considering the elastic deformation was irrelevant to the TWSMA pre-deformation, the pre-deformation stress (Δ*σ*) could be described as Δ*σ* = *σ*_s_ − *σ*_MR_, where *σ*_s_ was the maximum loading stress for each stress-strain curve in Figure 6. It could be seen that the restored strains of samples after unloading were almost less than 1% during cryogenic pre-deformation, which achieved the purpose of cryogenic pre-deformation under the martensite phase state. The lengths of the samples which were unloaded at low temperature and moved into room temperature were measured by the Vernier caliper. It was found that the samples with less than a 12% pre-deformation strain had an almost complete recovery, whereas the samples which underwent a pre-deformation strain of 12% and above still retained large unrecovered strains (no recovery).

**Figure 7:**
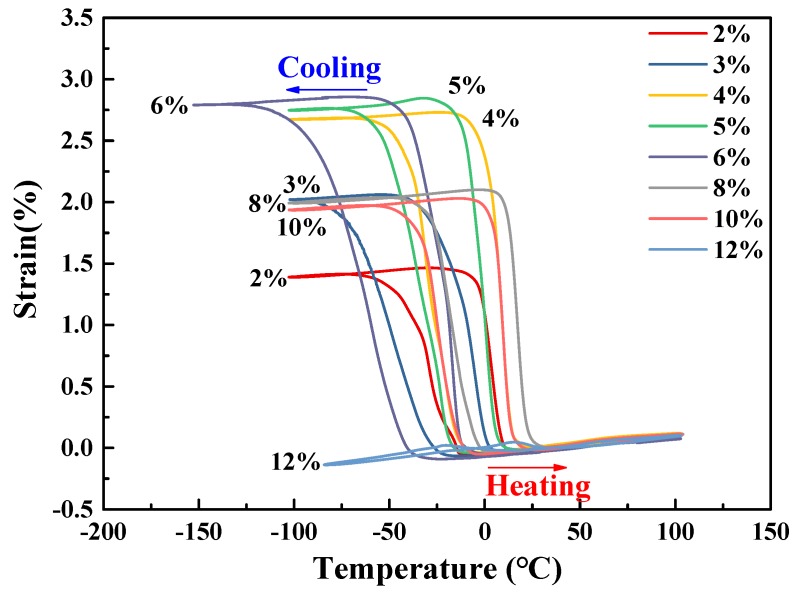
TWSME exhibited in thermal cycles after different cryogenic pre-deformations of columnar-grained Cu_71.7_Al_18.1_Mn_10.2_ alloy samples.

**Figure 8:**
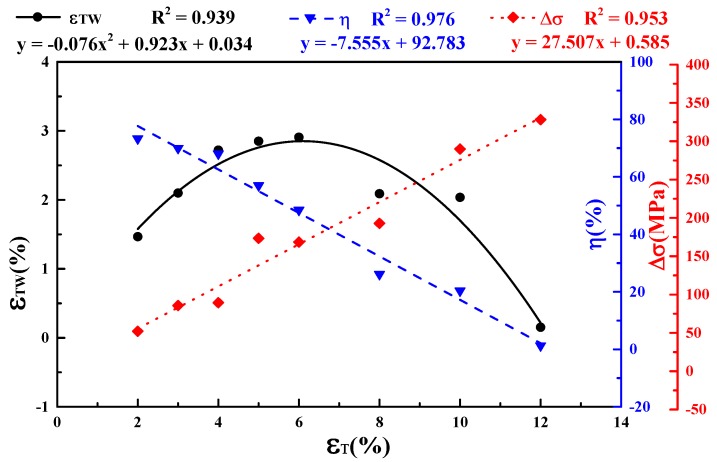
Effects of *ε*_T_ on the *ε*_TW_, Δσ and η of columnar-grained Cu_71.7_Al_18.1_Mn_10.2_ alloy samples.

Figure 7 shows macroscopic deformation induced by the transformation strain, i.e., TWSMA, during thermal cycles for all samples after different cryogenic pre-deformation deformations. The value of the transformation strain *ε*_TW_ is calculated by $\frac{\Delta L}{L_{0}}\times 100\%$, where Δ*L* is the average absolute value of expansion during the cooling process and of contraction during the heating process along the longitudinal direction of the samples. Figure 7 indicates that the samples elongated during cooling, whereas they contracted during heating. The variation of the strain induced by TWSME denoted as *ε*_TW_ are shown in Figure 8. It also shows that *ε*_TW_ increased with increasing *ε*_T_ to a maximum of 2.91% when *ε*_T_ = 6% and then decreased as shown by black dots in Figure 8. When the *ε*_T_ reached 12%, the *ε*_TW_ decreased to nearly zero, which indicated TWSME disappeared when undergoing a pre-deformation strain that was equal to or higher than 12%. The polynomial fitting (the black solid line in Figure 8) equation is $y=-0.076x^{2}+0.923x+0.034$, where y is *ε*_TW_, x is *ε*_T_, with a correlation coefficient *R*^2^ of 0.939. Similarly, the Δ*σ* and pre-deformation efficiency $\eta =\frac{\varepsilon_{\mathrm{TW}}}{\varepsilon_{T}}\times 100\%$ vs. *ε*_T_ were also plotted and linear fitted in Figure 8. The fittings equations for η (the blue dashed line in Figure 8) and Δσ (the red dotted line in Figure 8) function as *ε*_T_ being y=−7.555x+92.783 and y=27.507x+0.585 respectively, corresponding to *R*^2^ being 0.976 and 0.953, respectively. Δσ increased linearly with the increase of *ε*_T_, but η decreased linearly. Figure 8 indicates that a maximum *ε*_TW_ of −2.9% can be obtained where Δσ = 150–180 MPa and *ε*_T_ = 5–6%, corresponding to η of ~55.0%. 

**Figure 9:**
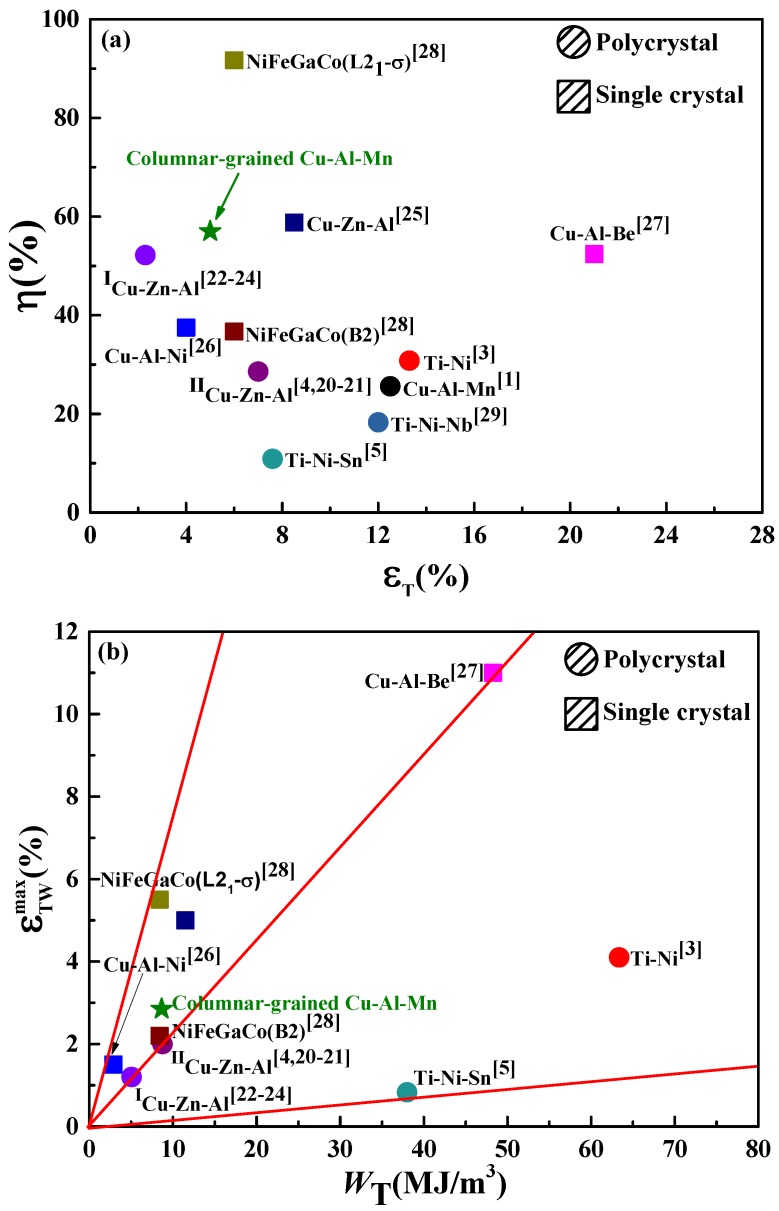
(**a**) Comparisons of η vs. *ε*_T_ and (**b**) $\varepsilon_{\mathrm{TW}}^{max}$ vs. *W*_T_ for different SMAs.

In Figure 9a,b, the values of η, the maximum *ε*_TW_ ($\varepsilon_{\mathrm{TW}}^{max}$) and the pre-deformation work (*W*_T_ = $\sigma_{s}$ × *ε*_T_) done by pre-deformation corresponding to the $\varepsilon_{\mathrm{TW}}^{max}$ from literature [20,21,22,23,24,25,26,27,28,29] and this work were compared. From Figure 9, it could be found that the single crystals showed higher η and $\varepsilon_{\mathrm{TW}}^{max}$ and lower *W*_T_ than their polycrystalline counterparts. The value of η for columnar-grained Cu_71.7_Al_18.1_Mn_10.2_ alloy was ~55.0% corresponding to ~3% *ε*_TW_. Comparatively, the η values were ~25.6% and 34.0% corresponding to the same *ε*_TW_ obtained by bending pre-deformation in ordinary polycrystalline Cu–Al–Mn alloys [1] and obtained by tensile deformation in the Ni–Ti alloy [3], respectively. The value of *W*_T_ for columnar-grained Cu_71.7_Al_18.1_Mn_10.2_ alloy was only ~3 MJ/m^3^, while for the Ti–Ni alloy, it was ~15 MJ/m^3^. Figure 9 indicates that the shape memory properties of columnar-grained Cu_71.7_Al_18.1_Mn_10.2_ alloy were better than ordinary polycrystalline Cu-based SMAs and reached a single crystal level. 

**Figure 10:**
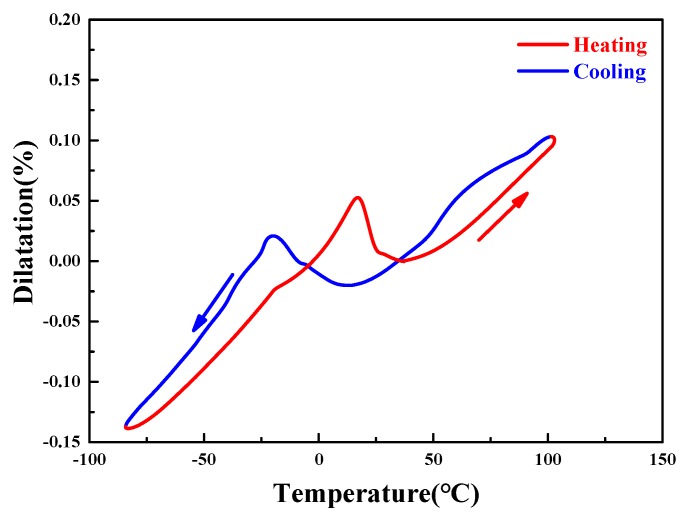
The thermal expansion measurement of the Cu_71.7_Al_18.1_Mn_10.2_ alloy sample without pre-deformation process.

For SMAs, the *ε*_TW_ was much larger than the thermal expansion. Figure 10 indicates the thermal expansion curve of a columnar-grained Cu_71.7_Al_18.1_Mn_10.2_ alloy sample without deformation pre-deformation. The thermal expansion coefficient was measured to be ~12.9 × 10^−6^/°C for both the austenite and martensite. It was seen that a near-linear dimensional expansion of ~0.2% was involved in transformation from −80 to 80 °C without deformation pre-deformation. Hence, when compared to the *ε*_TW_ mentioned above, the inherent thermal expansion of columnar-grained Cu_71.7_Al_18.1_Mn_10.2_ alloy could be ignored.

### 3.3. TWSME and Transformation Temperatures

The thermoelastic martensite played a vital role above room temperature after cryogenic pre-deformation in columnar-grained Cu_71.7_Al_18.1_Mn_10.2_ alloy samples. The cryogenic pre-deformation was attributed to the variants of self-accommodation and reorientation with an internal elastic energy ($\Delta E_{\mathrm{el}}$) and an irreversible energy ($\Delta E_{\mathrm{ir}}$), expressed as follows for the reversible thermoelastic martensitic transformation [3]:

(1) $$\;\Delta G=\Delta H^{A–M}-T\Delta S^{A–M}+\Delta E_{\mathrm{el}}^{A–M}+\Delta E_{\mathrm{ir}}^{A–M}\;$$

In this equation, the superscript “A–M” denotes the reversible transformation between the austensite and the martensite. The critical temperature for the reversible transformation can be determined at ΔG=0 as:

(2) $$\;T_{0}=\frac{\Delta H^{A–M}+\Delta E_{\mathrm{el}}^{A–M}+\Delta E_{\mathrm{ir}}^{A–M}}{\Delta S^{A–M}}\;$$

It was seen that *T*_0_ was directly influenced by $\Delta E_{\mathrm{el}}$ and $\Delta E_{\mathrm{ir}}$. $\Delta E_{\mathrm{el}}$ was defined as the elastic potential difference between the martensite and the austensite. As in the stress-strain curves shown in Figure 6, before stress yield, the elastic potential was stored as internal elastic strains caused by the formation of martensite variants. The $\Delta E_{\mathrm{el}}$ was expected to be dependent on the accommodation structure discrepancy of martensite variants formed by forward martensitic transformation on cooling.

The $\Delta E_{\mathrm{ir}}$ could have been caused by many factors. The first possibility was residual martensite due to cryogenic pre-deformation, which resulted in a macroscopically unrestored strain, even with the sample heated to a high temperature above the transformation temperature. The second possibility was defects in the sample, which obstructed the completion of the transformation. The third possibility was friction induced by phase interface and martensite variants during the reorientation of self-accommodation martensite variants induced by pre-deformation. It was difficult to clarify the effect mechanism of pre-deformation on the TWSME. However, Equation (2) implies that the variation of phase transformation temperature could be used to characterize the change of internal energy induced by pre-deformation.

**Figure 11:**
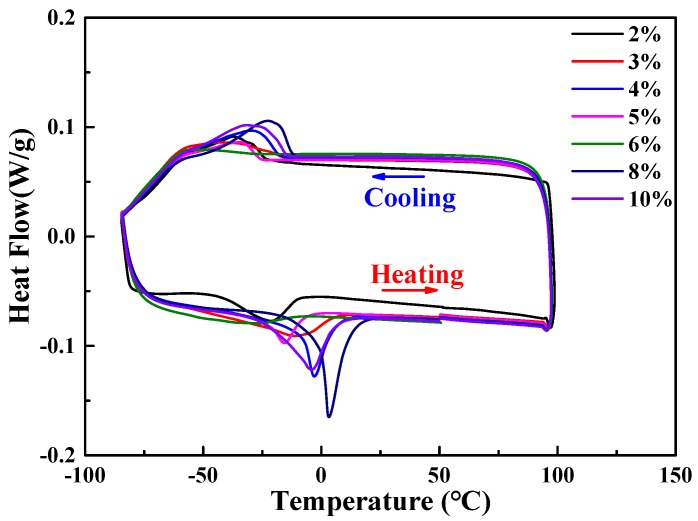
DSC measurements after different cryogenic pre-deformations.

**Figure 12:**
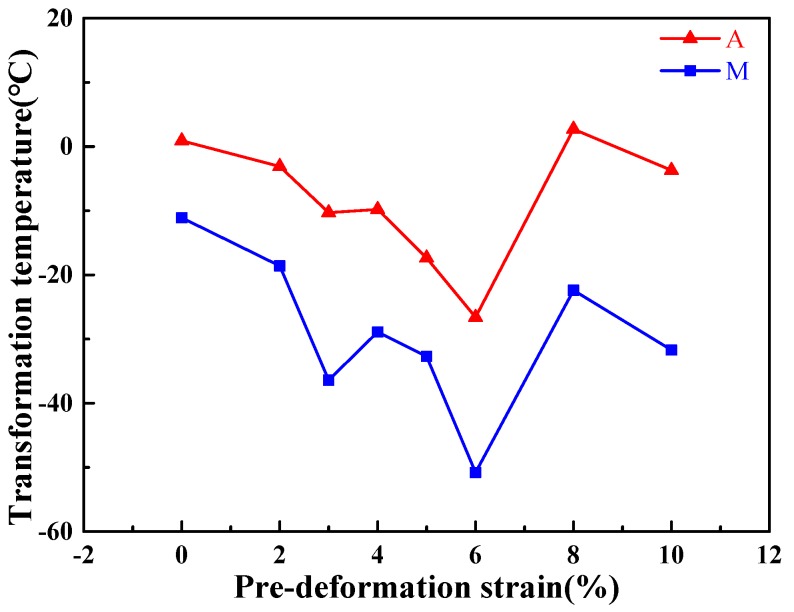
Transformation temperatures on DSC samples after different cryogenic pre-deformations. Note: *A* = (*A*_s_ + *A*_f_)/2; *M* = (*M*_s_ + *M*_f_)/2.

Figure 11 indicates that the DSC curves for the samples suffered cryogenic pre-deformation with different pre-deformation strains. Based on the DSC curves, variation of the transformation temperatures of the columnar-grained Cu_71.7_Al_18.1_Mn_10.2_ alloy samples with the increase of pre-deformation strain could be obtained and plotted in Figure 12. Figure 12 shows that the transformation temperatures of the columnar-grained Cu_71.7_Al_18.1_Mn_10.2_ alloy samples decreased at first and then increased with the increase of the pre-deformation strain. The change of transformation temperatures could be attributed to the internal stress created during cryogenic pre-deformation. When the sample was loaded with small strains during cryogenic pre-deformation, the reorientation of self-accommodation martensite variants became the dominative deformation mechanism. The internal stress of the sample decreased with the completion of reorientation. When the reorientation completely finished, the plastic deformation was induced by a dislocation slip mechanism, which would increase the internal stress by interaction of dislocations and other microstructure defects such as grain boundary and phase interface. Figure 12 shows that the lowest transformation temperature corresponds to a pre-deformation strain of 6%. Based on the above thermodynamic analysis, the lowest transformation meant the lowest phase transformation driving energy, i.e., the lowest resistance of phase transformation. Therefore, the largest TWSME could be obtained in the sample after a 6% pre-deformation strain. 

### 3.4. TWSME and Martensite Morphology

**Figure 13:**
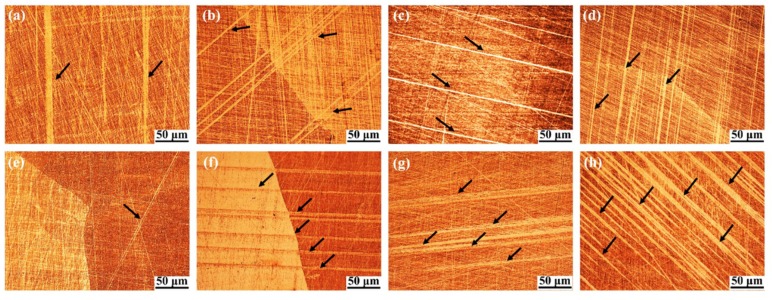
The morphology of martensite plates were observed at room temperature in the samples after different cryogenic pre-deformations; (**a**–**h**) were pre-deformation strains of ~2%, 3%, 4%, 5%, 6%, 8%, 10% and 12% respectively.

As shown in Figure 13, martensite plates in the grain and cross grain were not distributed in all of the grains of the Cu_71.7_Al_18.1_Mn_10.2_ samples after cryogenic pre-deformation at room temperature, which illustrated that martensites formed preferentially in some grains with little resistance during transformation. The martensite plates were thin when pre-deformation strain was ≤6% as shown in Figure 13a–e, while they became thicker when pre-deformation strain was over 6%, especially reaching ~12% as shown in Figure 13f–h. The observations of martensite morphology indicate that thin martensite plates show a tiny effect on TWSME, while thicker martensite plates degrade TWSME.

## 4. Summary

In this paper, we have investigated the relationship between pre-deformation strain and TWSME in the columnar-grained Cu_71.7_Al_18.1_Mn_10.2_ alloy. With the raising of pre-deformation strain, TWSME increased at first and then decreased, and the maximum transformation strain of 2.91% was measured under 6% pre-deformation strain. Meanwhile, the high pre-deformation efficiency of 57% and low applied pre-deformation work of ~3 MJ/m^3^ were found in the columnar-grained Cu_71.7_Al_18.1_Mn_10.2_ alloy, which indicated that the columnar-grained Cu-based SMAs show a better shape memory property than ordinary polycrystalline SMAs.
